# Targeting Integrin-Dependent Adhesion and Signaling with 3-Arylquinoline and 3-Aryl-2-Quinolone Derivatives: A new Class of Integrin Antagonists

**DOI:** 10.1371/journal.pone.0141205

**Published:** 2015-10-28

**Authors:** Sandrine Fiorucci, Xiaochen Lin, Karin Sadoul, Guy Fournet, Daniel Bouvard, Olga Vinogradova, Benoît Joseph, Marc R. Block

**Affiliations:** 1 Université Grenoble Alpes, Grenoble, France; 2 INSERM, Centre de recherche U823, Grenoble, France; 3 University of Connecticut, Storrs, United States of America; 4 Université Claude Bernard-Lyon 1, Lyon, France; Candiolo Cancer Institute, ITALY

## Abstract

We previously reported the anti-migratory function of 3-aryl-2-quinolone derivatives, chemically close to flavonoids (Joseph et al., 2002). Herein we show that 3-arylquinoline or 3-aryl-2-quinolone derivatives disrupt cell adhesion in a dose dependent and reversible manner yet antagonized by artificial integrin activation such as manganese. Relying on this anti-adhesive activity, a Structure-Activity Relationship (SAR) study was established on 20 different compounds to throw the bases of future optimization strategies. Active drugs efficiently inhibit platelet spreading, aggregation, and clot retraction, processes that rely on α_llb_β_3_ integrin activation and clustering. *In vitro* these derivatives interfere with β_3_ cytoplasmic tail interaction with kindlin-2 in pulldown assays albeit little effect was observed with pure proteins suggesting that the drugs may block an alternative integrin activation process that may not be directly related to kindlin recruitment. *Ex vivo*, these drugs blunt integrin signaling assayed using focal adhesion kinase auto-phosphorylation as a read-out. Hence, 3-arylquinoline and 3-aryl-2-quinolone series are a novel class of integrin activation and signaling antagonists.

## Introduction

Many physiological and pathological processes are largely dependent on cell adhesion to the surrounding extracellular matrix (ECM) [1]. Integrins, the main cell adhesion receptors, are transmembrane, heterodimeric proteins composed of a large extracellular domain that interacts with specific ECM proteins and a short cytoplasmic tail which recruits a complex and dynamic platform of proteins involved in both signaling and mechanical functions.

Integrins are central regulators of cell fate and have raised a great interest as therapeutic targets [2–5]. So far many of the twenty four known integrin hetero-dimers have been targeted due to their involvement in immunity [6], cancer [7], and processes such as platelet aggregation or angiogenesis [8]. On the other hand, α_v_β_3_ integrins [9] and α_2_/α_5_β_1_ [10] have recently been shown to bypass tyrosine kinase inhibitors treatments leading to resistances in cancer therapies. Some integrin inhibitors are undergoing late-stage clinical trials in cancer, inflammation, autoimmune disorders and thrombosis treatment. Most of integrin targeting compounds, including antagonist monoclonal antibodies, peptide derivatives and small molecule inhibitors, were designed on a ligand-based strategy. They mimic all or part of the binding domain of integrin substrates, often recognized through their RGD motif. Such molecules act as competitors and efficiently prevent integrin mediated cell attachment or platelet aggregation. Unfortunately though, they also act as partial agonists of integrin signaling [11], leading to significant side effects that have, so far, reduced the use of the related therapeutic strategies [12–14]. For platelets, the use of intravenous α_IIb_β_3_ antagonists has been superseded by the combination of aspirin and a P2Y12 inhibitor due to decreased bleeding risk and lower cost. Finally, treatments with integrin antagonists often result in modification of the integrin expression pattern and drug resistance, hence putting forward the need of inhibitors with multiple targets [15].

Extracellular stimuli lead to a direct interaction between the β-integrin cytoplasmic tail and the FERM-like domain of talin, which is regarded as a major integrin activator [16, 17]. The interaction of integrin cytosolic domain with talin unclasps the complex between α and β subunit tails [18, 19], triggering conformational changes along transmembrane and extracellular integrin domains. This rearrangement allows the shift between the integrin “low affinity state”, where the extracellular domain is folded toward the membrane resulting in access hindrance for extracellular ligands, to an extended, “high affinity state" where the interaction with the extracellular matrix is favored. However, talin alone is not sufficient to trigger full integrin activation. Kindlins can also interact directly with the integrin cytoplasmic tail through a FERM-like domain [20] on a NPxY motif distinct from the talin binding site and act as a talin cofactors required for effective integrin activation. Finally, it has recently been proposed that in platelets, talin triggers the switch to the high affinity state of α_IIb_β_3_ while kindlin-3 favors integrin clustering [21], a state leading to an irreversible aggregation.

Need to develop new strategies for integrin-based therapies with limited side effects will mainly rely on the design of antagonists that block both integrin/extracellular matrix interaction and integrin signaling. Consistent with this view, it has been recently reported that blocking the interaction between the integrin α_4_ subunit and paxillin with a small membrane permeable molecule may be used in anti-inflammatory treatment [22]. This inhibition abrogates α_4_-integrin mediated responses in T-cells while maintaining integrin-independent responses.

Herein, we describe potent integrin inhibitors with a 3-arylquinoline or a 3-aryl-2-quinolone scaffold that inhibit integrin mediated cell adhesion and integrin signaling (See supplemental information section for structural description of the molecules). These compounds, that share similarities with flavonoids, were initially described as cell migration inhibitors [23]. Indeed, 3-aryl-2-quinoline BJINT006, the lead compound of this study, strongly impacted focal adhesions (FA) assembly and dynamics in a reversible and dose-dependent manner. Since platelet activation is a physiological process that is strictly dependent on the integrin α_IIb_β_3_ [24], platelets were treated with BJINT small molecules to assess their putative anti-thrombotic potential. As expected, the treated platelets showed impaired spreading and aggregation and were unable to trigger efficient clot retraction. Finally, *ex vivo*, these drugs blunt outside-in integrin signaling. Altogether, our results showed that 3-Arylquinoline and 3-aryl-2-quinolone derivatives are integrin antagonists that might be used for integrin based therapy such as anti-thrombotic agents.

## Materials and Methods

### Reagents and antibodies

Fibronectin was purified from bovine plasma as described previously [25], rat tail collagen I was purchased from Becton Dickinson (Pont-de-Claix, France), vitronectin from Life Science Invitrogen (Saint-Aubin, France) and fibrinogen from EMD Millipore (Molsheim, France). Poly-lysine was obtained from Sigma-Aldrich (L'isle-d'Abeau, France). Monoclonal antibodies raised against Kindlin-2 (clone 3A3) and talin (clone TA205) were purchased from EMD-Millipore. β_3_ integrin mAb was obtained from Emfret (Eibelstadt, Germany). Various Alexa-488 conjugated antibodies were obtained from Invitrogen and HRP-coupled antibodies from Biorad (Marnes-la-Coquette, France). Blebbistatin was purchased from EMD Millipore, TRITC-phalloidin and Thrombin from Sigma-Aldrich and collagen for platelet aggregation assays from Helena Biosciences.

### Plasmids and cell lines

Talin head was inserted in pDsRed C1 plasmid (Clonetech, Saint-Germain-en-Laye, France) and transfected in pre-osteoblasts using Lipofectamine 2000 (Invitrogen) according to provider procedure. GFP-Kindlin-2 expressing pre-osteoblasts were obtained from newborn mice sacrificed by decapitation as previously described [26]. Kindlin^fl/fl^ preosteoblasts were isolated from corresponding mouse strain generated by EMMA and transfected par ERT2Cre plasmid [27]. FAK -/- pre-osteoblasts were prepared from FAK^fl/fl^ mice, immortalized with SV40 large T antigen followed by gene deletion after infection with adenoviruses expressing the Cre recombinase.

In France since February 2013, according to the European Directive 2010–63, research using animal models is subject to authorization from the Ministry of Research. Each project authorization includes ethics opinion issued by the ethics committee for animal experiments joined by the animal facility. The ethics committee of Grenoble, ComEth-Grenoble is registered with the National Committee of Reflection on Ethical Animal Experiments of the Ministry of Research under number 12 (CEEA No. 12). The main task of the committee is to issue reasoned opinion on the ethics of experimental projects proposed by the experimenters. The committee has validated this project

### Cell immunostaining

Cells were fixed with 4% PFA, platelets with 4% neutral formalin (Sigma-Aldrich). They were permeabilized with 0.2% Triton X100 in PBS and incubated with appropriate primary antibodies in PBS- supplemented with 10% goat serum- 0.1% Tween. After 3 washes, the coverslips were incubated with appropriate secondary antibodies. Cells were mounted in Mowiol solution and imaged on an inverted microscope (Axioimager, Carl Zeiss S.A.S., LePecq, France).

### Cell spreading assay

Cell adhesion was assayed as previously described (Yan, 2008). Briefly, 96-wells plates were coated with 5 μg/mL of fibronectin overnight. Washed twice with PBS, the wells were then blocked in 1% BSA for 1 h at 37°C. Cells (1.10^4^/well) in suspension were incubated with or without BJINT molecule for half an hour at 37°C before being allowed to adhere for 30 min. Cells were washed with PBS and fixed in 10% methanol– 10% acetic acid before staining in 0.5% Crystal Violet (Sigma Aldrich)- 10% methanol for 10 min at room temperature. Wells were rinsed three times before dissolving the dye in 10% acetic acid at 37°C for 15 min. Absorbance at 620 nm was read on an Anthos AD3405 plate reader (Beckman Coulter, Villepinte, France).

### Adhesion inhibition assay

The ability of 3-arylquinoline or 3-aryl-2-quinolone derivatives to detach spread cells was assayed by modifying the spreading assay protocol. Plates were coated as described above. Cells were allowed to spread for 1 h at 37°C then the medium was removed and replaced by medium containing the tested molecule at the desired concentration. Cells were treated for 1 h then washed before fixation and coloration as described above.

### Cell spreading on various substrates

Coverslips were coated either with 10 μg/mL fibronectin or vitronectin in PBS at 4°C overnight or with 20 μg/mL collagen for 1 h at 37°C in 0.2M acetic acid. The coated coverslips are then blocked with 1% BSA for 1 h at 4°C. Cells expressing GFP-kindlin-2 were incubated in PBS 5% BSA for 45 min at 37°C before being allowed to spread on the coated coverslips overnight in DMEM with 10% fibronectin free FCS. Medium is then replaced with medium containing the tested molecule or the corresponding volume of DMSO. The cells were incubated 1 h at 37°C before prior fixation.

### Spreading on poly-lysine versus fibronectin

Cells spread on fibronectin (integrin dependent) or poly-lysine (integrin independent) 35-mm-diameter cell culture dishes were coated overnight at 4°C with 10 μg/mL fibronectin or 100 μg/mL poly-lysine. Fibronectin coated dishes were then blocked with 1% BSA while poly-lysine coating was continued for 1 h at 37°C. Then the PLL coated dishes were dried for at least 3 hours before use. Cells were incubated in 5% BSA for 1 h at 37°C before being centrifuged and resuspended in DMEM with 10% fibronectin free FCS and allowed to spread on dishes for 2 h. Medium was then removed and replaced with DMEM with fibronectin free serum containing BJINT006 at 12.5 or 25 μM. Cells were incubated for 1 h at 37°C before fixation. Remaining cells were then manually counted on 15 microscope fields.

### Live/dead cell toxicity assays

Cells in D-MEM supplemented with 10% fetal calf serum were incubated with 50 μM of BJINT molecules or DMSO for 1 h at 37°C. 10^6^ cells were centrifuged and washed two times with PBS then resuspended with 200 μL of PBS. Then 1 μL of freshly prepared 1 μM Calcein AM solution in PBS was added. Incubation was performed for 45 min at 37°C. Then 5 μL of propidium iodide solution (10 mg/mL) were added. After homogenization 0.5 mL of cold PBS was added and cells were immediately analyzed by FACS (FL1 vs FL3 channels).

### Preparation of human platelet-rich plasma (PRP) and Platelet-poor plasma (PPP)

Human platelet-rich plasma concentrates from anonymous donors were obtained from the French national blood bank, Grenoble Branch, and used accordingly to the European rules and approved by Grenoble University Ethical committee. Human PRP and PPP were prepared as previously described [28]. Briefly, non-therapeutic buffy coats were diluted with an equal volume of PBS and centrifuged 10 min at 400g at RT. The upper phase corresponding to the PRP was collected, and a part of the lower erythrocyte rich fraction is kept for the clot retraction assay, as mentioned below. PPP was obtained by collecting the supernatant after centrifugation of PRP for 5 min at 2200g.

### Platelet spreading assay

Platelets resuspended in PBS were preincubated for 30 min with BJINT006 at the tested concentrations. A number of 1.10^6^ platelets/well/400 μL were then seeded into 24-wells plates containing fibrinogen- or collagen-coated coverslips, centrifuged (3 min, 600g, RT) and placed in an incubator for 30 min before fixation.

### Platelet aggregation assay

For aggregation assays PRP was adjusted to a platelet concentration of 3x10^8^/mL with PPP and different concentrations of BJINT drugs or vehicle were added and preincubated for 30 min. Platelet aggregation was induced by adding 50 uL of collagen (20 μg/mL in 0.9% NaCl) or ADP (10 μg/mL) to 150 μL PRP and evaluated using an APACT 4004/LABiTec aggregometer.

### Clot retraction assay

PRP was adjusted to 3x10^8^ platelets/mL with PPP, 400 μL were pipetted into an aggregation tube and 2 μL of an erythrocyte rich fraction (see PRP preparation) was added for color contrast. After an initial 30 min incubation at 37°C coagulation was induced by adding 4 μL of thrombin (20 U/mL, 0.1% BSA) and mixing with a plastic inoculation loop. After 10 min the clots were removed, and placed into new tubes containing 400 μL PBS. Pictures were taken for visual inspection and the remaining extruded serum volume was measured for quantitative evaluation of clot retraction.

### Expression and purification

Cloning, expression, and purification the β_3_ cytoplasmic tail in non- and mono-phosphorylated forms have been described previously [18, 29]. To produce ^15^N-labeled proteins, cells were grown in M9 minimal medium containing ^15^NH4Cl (1.1 g/L) as the sole source of nitrogen. Expression of GST tagged β_1_, β_3_, F2/F3 talin domain, or kindlin-2 FERM domains were purified by affinity on Glutathion-Sepharose 4B (GE heathcare). Biotinylation of purified proteins was carried out using EZ link NHS kit from ThermoScientific according to the manufacturer's instructions.

### Pull-down assays

GST β_1_ and β_3_ cytoplasmic tails were expressed in the BL21-CodonPlus–RIL E. coli strain. Bacteria were disrupted in a buffer made of 50 mM TRIS Cl pH 7.5, 1% (w:v) Triton X100; 150 mM NaCl, 5 mM MgCl_2_, 2 mM DTT and anti-protease mix (Roche) by sonication. The lysate was clarified by centrifugation at 14000 rpm in a JA20 rotor (Beckman) and used immediately or frozen in liquid nitrogen and stored at -80°C. Glutathione coupled Sepharose beads (160 μL of bead suspension) were washed with the bacteria lysis buffer, mixed with 1 mL of bacterial lysate, and incubated overnight at 4°C. Beads were washed 3 times with cell lysis buffer and resuspended in 1 mL of this buffer before use.

Pre-osteoblast cells (90% confluent) expressing either GFP kindlin-2, or DsRed talin head in 10 cm Petri dishes were washed with 10mL/dish of cold PBS, then lysed by 1 mL/dish with cell lysis buffer (10 mM PIPES pH 6.8, NaCl 100 mM, Na_2_VO_4_ 1 mM, Na_2_PO_4_,7H_2_O 50 mM, 0.1% (w:v) Deoxycholate, Sucrose 150 mM, 0.5% (w:v) Triton X100, anti-protease mix). The lysate was clarified by centrifugation at 13600 rpm for 15 min.

Beads and cell lysates were pre-incubated separately with 50 mM of BJINT derivatives or vehicle for 1 h with agitation at 4°C, then 250 μL of bead suspension were mixed with 0.6 mL of cell lysate and incubation was continued for 4 h at 4°C. After 3 washes in cell lysis buffer the beads were drained and mixed with 15 μL of Laemli sample buffer and elutes proteins were analysed by SDS-PAGE and Western blotting with anti kindlin or anti talin head primary antibodies.

### Solid phase binding assays

GST tagged recombinant proteins were expressed in the BL21-CodonPlus–RIL E. coli stain, purified on glutathione-Sepharose 4B beads according to classical procedures, frozen in liquid nitrogen, and stored at -80°C. GST β_1_ and β_3_ cytoplasmic tails were biotinylated using the EZlink SulfoNHS biotinylation kit (Themo Scientific) according to the manufacturer instruction.

All stages were carried out at room temperature. 96 wells plates (Maxisorp Nunc) were coated with 100 μL/well of 10 μg/mL of GST, GST-talin-F2/F3 domain, or GST kindlin-2-FERM domain for 1 h. The wells were post coated with 200 μL/well of PBS supplemented with 3% (w:v) BSA for another hour. Then GST-β integrin tails in the cell lysis buffer (100 μL/well) were introduced at increasing concentrations and let to incubate 1 h. After 3 washes with PBS (200 μL/well), 100 μL/well of streptavidin HRP ELISA grade (BioRad) in PBS 3% BSA were added and incubated for 1 h. Finally after 3 more washes with PBS, integrin tail detection was achieved using an ABTS solution (Vectastain) according to the manufacturer instructions and absorbance was measured at 405 nm. Coating with plain GST allowed us to determine the non-specific binding and this signal was subtracted from the signals obtained with GST-talin or GST-kindlin-2 domains. It is noteworthy that specific binding was only significant in the presence of non-ionic detergent.

## Results

### FA disassembly is triggered in a dose dependent and reversible manner by BJINT006 and BJINT011 compounds, but not by BJINT020

**Fig 1:**
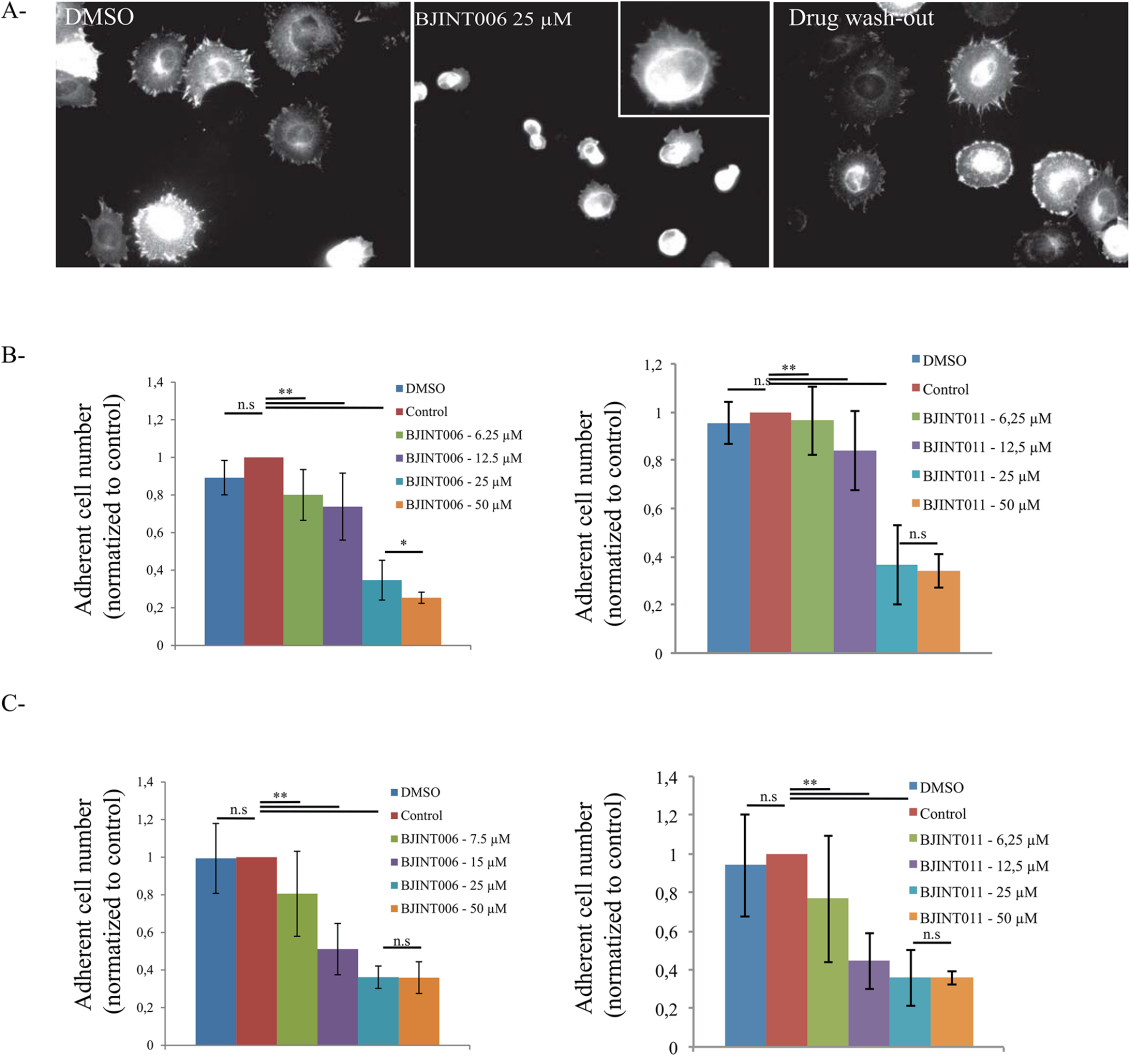
BJINT derivatives promote focal adhesion disassembly and cell detachment in a reversible and dose dependent manner. (A) Suppression of cell spreading under BJINT006 treatment. Pre-osteoblasts expressing GFPkindlin-2 were spread on fibronectin coated coverslips (5 μg/mL) for 1 h 30 at 37°C before adding the BJINT drugs and incubation was continued for 1 h. (left and central panels) Reversibility of BJINT006 after 1 hour drug wash-out (right panel). (B) Cells were treated with increasing BJINT006 and 011 concentrations and adhesion was compared to untreated cells (control) or cells treated with vehicle (DMSO). Cells were spread for 1h30, and then treated for 1h. After fixation, they were stained with Crystal Violet (0.5% w:v) and optical density was read at 620 nm. (C) Cells in suspension were treated for 1h at 37°C then let to attach and spread on fibronectin coated dishes for 1 h 30. They were compared to cells treated with vehicle (DMSO) or untreated (control). Adherent cell numbers were estimated as described above.

Integrins are important receptors involved in cell migration by orchestrating the clustering of proteins at or near the plasma membrane. We generated mesenchymal cell line (pre-osteoblasts) expressing an important integrin regulator, kindlin-2, fused to eGFP to dynamically monitor adhesive structures such as FAs or focal complexes. Immunofluorescence labeling using a monoclonal anti-kindlin-2 antibody recognizing both endogenous and exogenous kindlins shows a perfect co-localization, indicating that the distribution of both proteins was identical (S1 Fig). To improve FA assembly, the cells were seeded on fibronectin coated coverslips and allowed to spread for 90 minutes in presence of DMSO, the drug vehicle. Under these conditions the cells were nicely spread and displayed typical peripheral focal adhesions. However, addition of BJINT006 to the medium triggered cell rounding up at the lowest concentrations tested, along with a clear kindlin-2 delocalization from FAs (Fig 1A). Increasing the drug concentration finally resulted in a dose dependent cell detachment which was estimated using Crystal Violet staining as described under Materials and Methods. Similar results were observed using 3-aryl-2-quinolone BJINT 011 (Fig 1B), a closely related drug with the lateral side chain positioned differently on the molecule backbone, while in the absence of this lateral side chain on 3-aryl-2-quinolone BJINT020, the molecule exhibited no effect on cells even when used at the high concentration of 50 μM (not shown). To rule out any possible artifacts due to drug toxicity, BJINT treated cells were subjected to Calcein AM/propidium iodide staining. FACS analyses revealed that cell viability was better than 95% under all experimental conditions (S2 Fig).

Having shown that the molecules were able to interfere with focal adhesion stability we asked whether it could prevent cell adhesion. Cells were first incubated in suspension with increasing drug concentrations and then let to attach and spread for 90 minutes. Quantification of the number of cells attached to the plates indicated that the compounds 006 and 011 not only triggered FA disassembly but were also able to prevent their formation within the same concentration range (Fig 1C).

To assess whether the molecules action was irreversible or not, medium containing BJINT006 was removed and the cells were extensively washed before being allowed to spread for 1 hour. After the drug wash-out, the cells were able to assemble FAs again, showing that BJINT006 inhibitory activity was reversible and that the drug is likely nontoxic during the time course of incubation (Fig 1A). In addition we observed that the BJINT006 dependent inhibition of adhesion was independent of the ECM protein used to coat coverslips, since cell rounding up and detachment was also observed on either type I collagen or vitronectin (S3 Fig). This lack of specificity suggested that the drugs target a component or regulatory mechanism common to all adhesive structures, thus are likely to act intra-cellularly.

### The activity of BJINT family members is closely related to their structure

**Fig 2:**
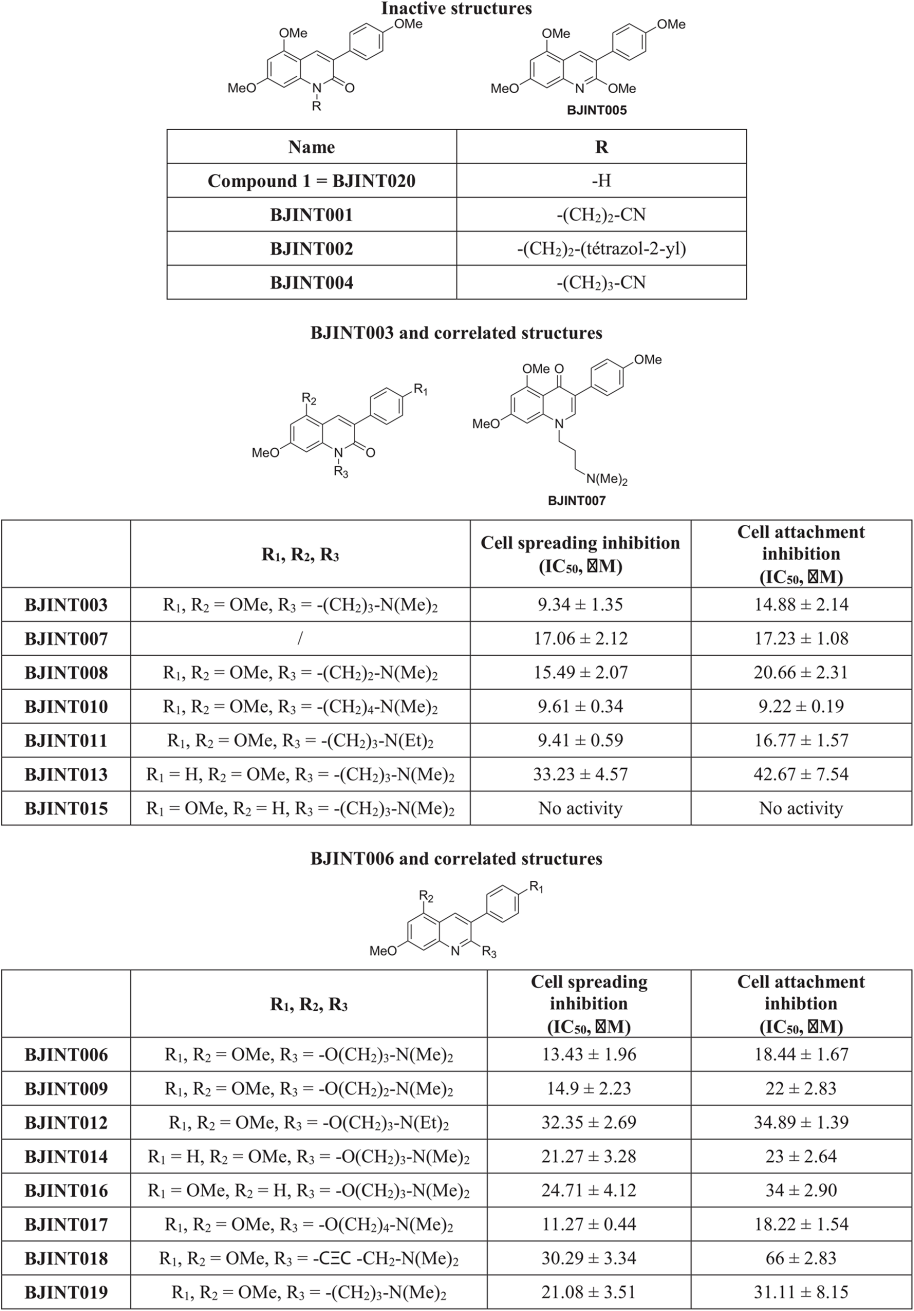
The activity of BJINT family members is closely related to their structure. Structure-activity relationship of 3-arylquinolines and 3-aryl-2-quinolones on cell spreading and attachment. BJINT003, BJINT006 and derivatives impair cell spreading and attachment mediated by focal adhesions. The activities have been estimated using at least three independent experiments where cells have been spread on fibronectin, fixed and stained with Crystal Violet.

To address the structure-activity relationship of 3-arylquinoline and 3-aryl-2-quinolone derivatives, compounds with modifications on their lateral positions were synthesized according to the strategy described in (S4 Fig). Twenty different compounds were tested using the adhesion assay described above. It appeared that the presence of a tertiary amine on R3 is required for BJINT family members to impede cell adhesion (Fig 2). Higher steric occupation around the amine may alter molecule activity depending on the lateral chain attachment to the backbone. Modification of the lateral chain length has little effect on the compound activity whereas increasing chain rigidity (BJINT018) seems to impair its activity, as does the suppression of R_1_ and R_2_ methoxy- substitutions. Whether longer chains can be introduced at these positions has yet to be investigated. This opportunity may raise the perspective of coupling the molecule to a fluorochrome or another relevant tool to further study the activity of the compounds.

Therefore, further key experiments were carried out with BJINT006 and BJINT011 as active compounds and BJINT020 as negative control. The structural differences in these two active molecules allowed assessing the importance of the lateral chain positioning on the main backbone of the molecule.

### 3-Arylquinoline and 3-aryl-2-quinolone derivatives inhibit FA assembly independently on the integrin Src/FAK signaling axis and cell contractility

**Fig 3:**
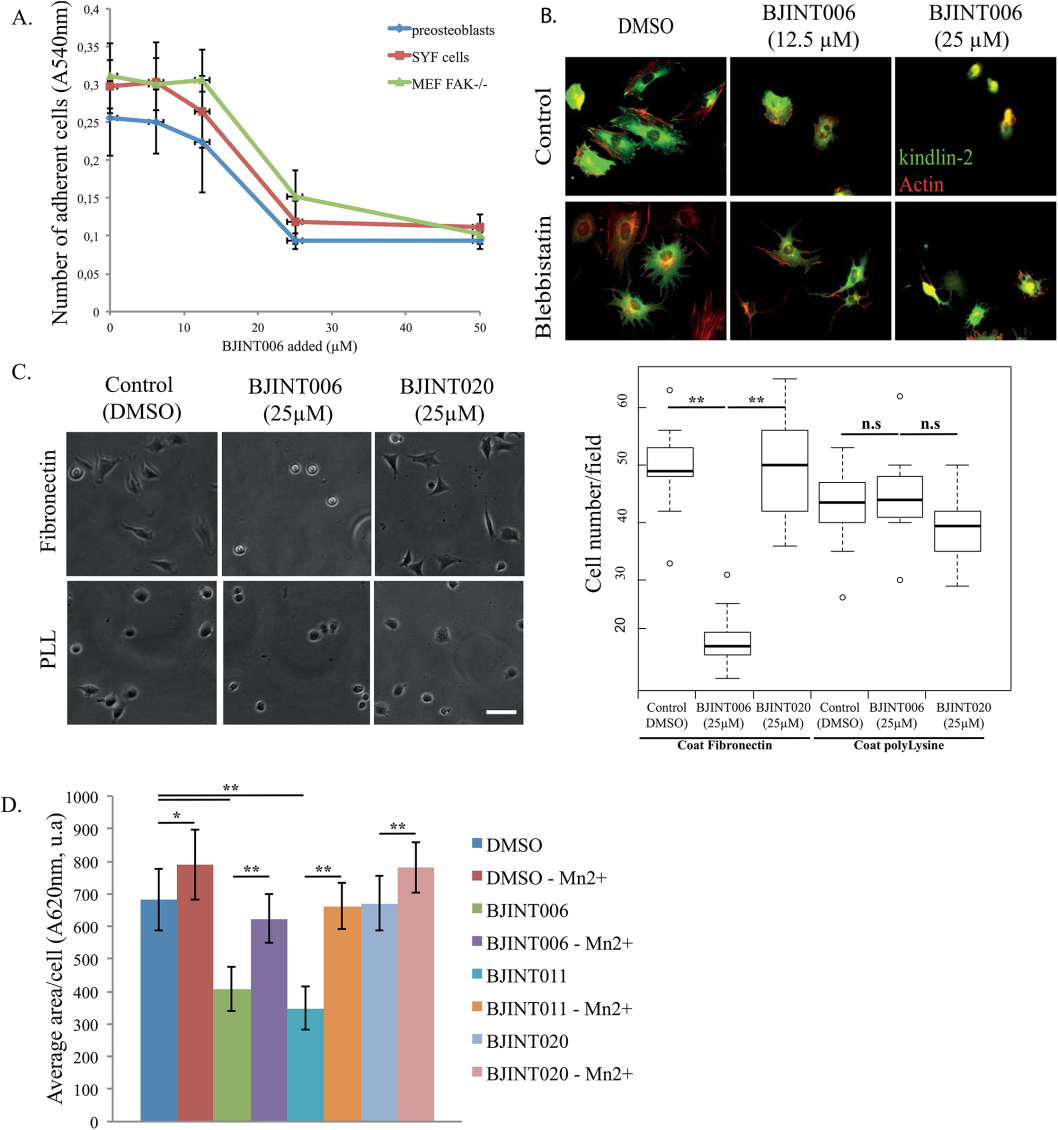
BJINT derivative inhibition of cell adhesion is integrin dependent. (A) Pre-osteoblasts, FAK^-/-^ MEFs, or MEFs devoid of Src, Yes, Fyn kinase expression (SYF cells) were allowed to spread for 1 h 30 on fibronectin coated multi-well plates in presence of increasing concentrations of BJINT006. Adhesion was inhibited similarly in all cell lines. (B) Pre-osteoblasts expressing GFP-kindlin2 spread for 1 h on fibronectin coated coverslips were treated with 10 μM Blebbistatin. BJINT006 at 12.5 or 25 μM in DMSO was then added directly into the cell medium and cell culture was continued for 2 h. Cells were fixed in 4% PFA. Cell contractility inhibition by Blebbistatin does not prevent cell rounding up after BJINT006 treatment. (C) Cells were seeded on fibronectin or poly-lysine for 2 h before 1 h incubation with BJINT006, 011, or 020. The cell adhesion on poly-lysine was not significantly modified by BJINT treatment in contrast to cells spread on fibronectin. (D) MnCl_2_, an artificial activator of integrins was introduced into the cell culture medium at the concentration of 1 μM prior addition of BJINT derivatives. It efficiently prevents cell adhesion inhibition by BJINT006 and 011.

FA disassembly caused by 3-arylquinoline and 3-aryl-2-quinolone derivatives may be of direct or indirect nature. The effect due to impairment of function or recruitment of the structural components, such as integrin receptors themselves, or linkers to cytoskeleton, including talins, kindlins, vinculin, is considered direct. The indirect action might occur through the perturbation of integrin signaling pathways, which include the activation state of Src-family kinases. On SYF cells (lacking Src, Fyn and Yes expression) as well as on FAK^-/-^ immortalized pre-osteoblasts, addition of BJINT 006 resulted in cell detachment (Fig 3A). This rules out any indirect effect in the drug action relying on FAK and Src-family kinases, two major actors in integrin signaling.

Along with adhesive structures, the actin cytoskeleton and cell contractility machinery are key players of cell migration [30]. Reciprocally, cell contractility dramatically impacts on adhesive structure assembly and patterning. To test whether BJINT006 adhesion inhibition depends on contractility, we treated GFP-kindlin-2 expressing pre-osteoblasts with a combination of BJINT006 and Blebbistatin, a specific myosin-II inhibitor [31], (Fig 3B). Blebbistatin at 10 μM was added to cells during their spreading and BJINT006 was added 2 h later. When cell contractility was inhibited with Blebbistatin, the cells displayed a greater projected area compared to untreated cells. However, BJINT006 treatment for two hours still resulted in cell rounding up, suggesting that myosin II is not the primary target of the drug and that the molecule is active even when integrin clustering cannot be triggered by inner tension.

### Integrin engagement in cell adhesion is necessary for BJINT full activity

Next, we wondered whether integrins or associated proteins might be directly targeted by the molecule. Indeed, if the protein platform, recruited around integrin cytoplasmic tails, or the integrins themselves were the molecular target of BJINT drugs, these compounds should be ineffective on cell adhesion when this latter process does not require integrin engagement with the ECM substrate. To address this question, we studied BJINT006 and BJINT020 adhesion inhibition on cells spread on fibronectin and compared it to cells attached to poly-lysine, a substrate known to support cell adhesion in a non-specific, integrin-independent manner [32]. When seeded on poly-lysine, the cells, although being adherent, did not spread on the substrate. Neither BJINT006 nor BJINT020 addition at the concentration of 25 μM significantly affected cell attachment to poly-lysine, while cell attachment on fibronectin was reduced by more than 90% by BJINT006 (Fig 3C). Consistent with the results described above, addition of BJINT020 did not significantly impair cell adhesion on both substrates. These results strongly suggested that the drugs specifically impaired integrin related adhesive structures. Specificity was further confirmed by the Mn^2+^ artificial switch of the integrins to the high affinity state that antagonized both BJINT006 and BJINT011 action (Fig 3D), while BJINT020 was once again ineffective on cell adhesion, independent on the experimental conditions used. These latter results suggested that the adhesion defect observed upon drug treatment was due to a lack of integrin activation.

### Platelet activation processes are impaired upon BJINT006/011 treatment

**Fig 4:**
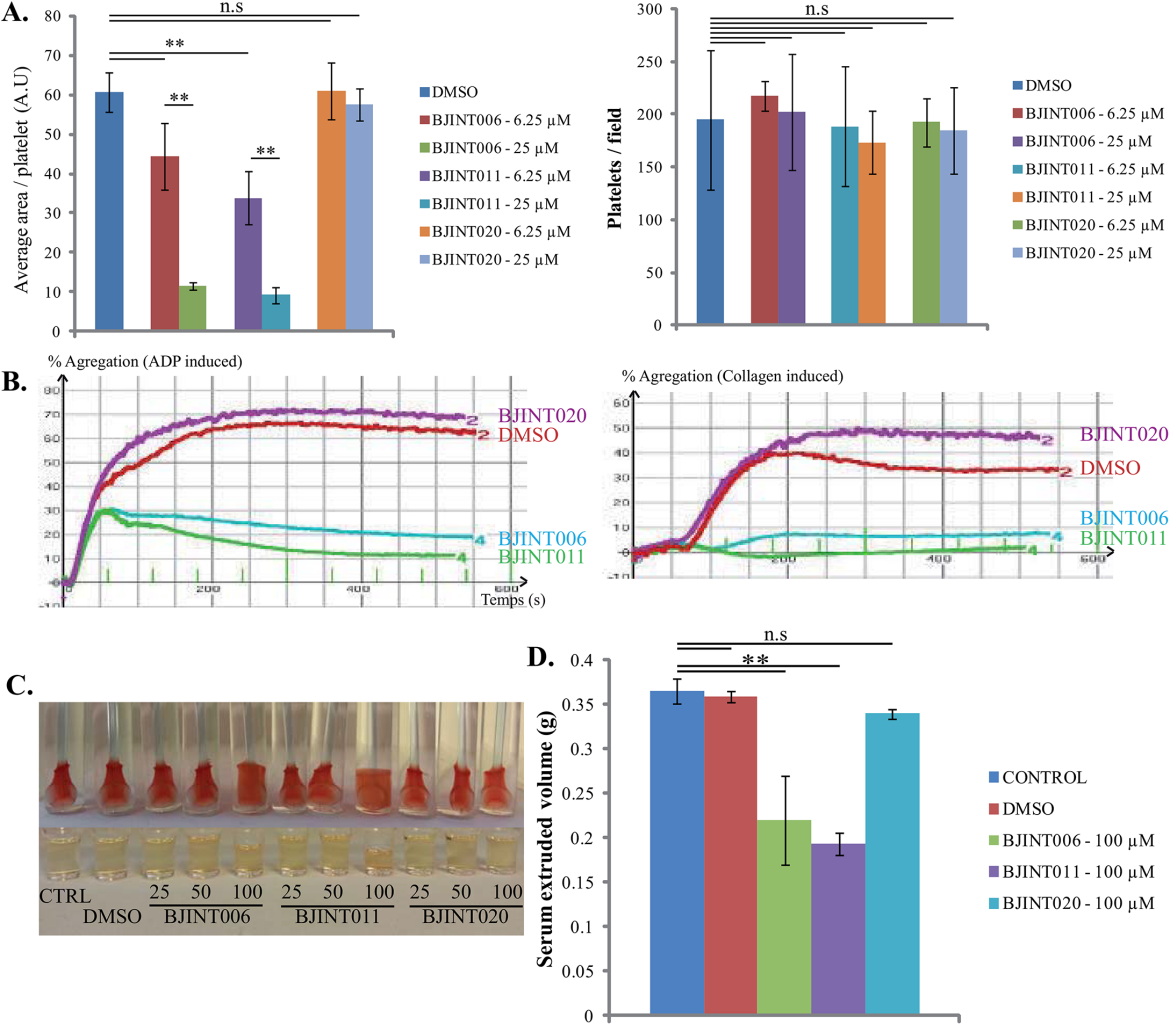
BJINT006 and 011 inhibit α_IIb_β_3_ mediated platelet activation related processes. (A) Platelet adhesion and spreading on collagen were carried out as described under Materials and Methods. Quantification of spreading and adhesion upon BJINT006, 011, and 020 treatments is shown on left and right panels, respectively. (B) Collagen- and ADP -induced aggregation kinetics are shown. Aggregation assays are carried out in the presence or absence of BJINT derivatives (50μM). (C) Clot retraction is induced by thrombin addition to PRP as described under Materials and Methods. BJINT006, 011 but not BJINT020, prevents proper clot retraction at the concentration of 100 μM. (D) The extruded serum volume was measured 10 minutes after thrombin addition to quantify clot retraction.

To further confirm that bioactive BJINT components inhibit integrin activation and/or clustering, we investigate the drug action on physiological and well characterized processes, namely platelet spreading, clot retraction, and platelet aggregation, that are largely dependent on α_IIb_β_3_ activation. Indeed, the switch from low to high affinity states is required for platelets to spread and aggregate. Furthermore, α_IIb_β_3_ provides the physical link between the platelet cytoskeleton and the fibrin network, which is essential for clot retraction. Therefore, we first checked whether BJINT006, 011 and 020 were able to prevent platelet spreading on fibrinogen. Similarly to what was found with pre-osteoblasts, platelet spreading was impaired by BJINT006 and 011 in a dose dependent manner but not by 020 (Fig 4A left panel). However, platelets adhesion was not affected by the drug, consistent with the known ability of α_IIb_β_3_ to interact with immobilized fibrinogen independently of integrin activation (Fig 4A right panel).

Platelet aggregation requires α_IIb_β_3_ linkage with fibrinogen. Indeed, in the presence of BJINT006 and BJINT011, collagen-induced platelet aggregation was completely abolished (Fig 4B right panel). This result clearly fits with experimental data showing that β_1_ knock out completely abolished aggregation in response to soluble collagen [33]. When aggregation was induced with ADP (Fig 4B left panel), the initial aggregation was normal but limited and platelets could not sustain aggregation over time suggesting that the secretory phase of aggregation did not occur. BJINT molecules are hydrophobic and must undergo a phase partitioning between membranes and cytosol as a prerequisite of their action. Therefore BJINT pharmacodynamics are likely slow. Consistent with this view, a 15 min preincubation of platelets with the drugs enable us to block aggregation to a similar level but with a twofold lower drug concentration (200 to 100 μM). Compared to ADP induction, collagen induction of platelet aggregation is slower and may provide sufficient time for the drugs to act. Finally, we studied the ability of treated platelets to achieve clot retraction which arises from the transmission of acto-myosin forces to the fibrin network through activated α_IIb_β_3_. At high concentrations of BJINT006 and 011 but not BJINT020 (100 μM), the clots fail to retract (Figs 4C and 2D). Altogether, these experiments showed that, as expected for an antagonist of integrin activation, BJINT006 and 011 exhibited a strong inhibition of platelet aggregation and are potential anti-thrombotic agents.

### BJINT derivatives inhibit integrin activation independently of kindlin and talin recruitment

**Fig 5:**
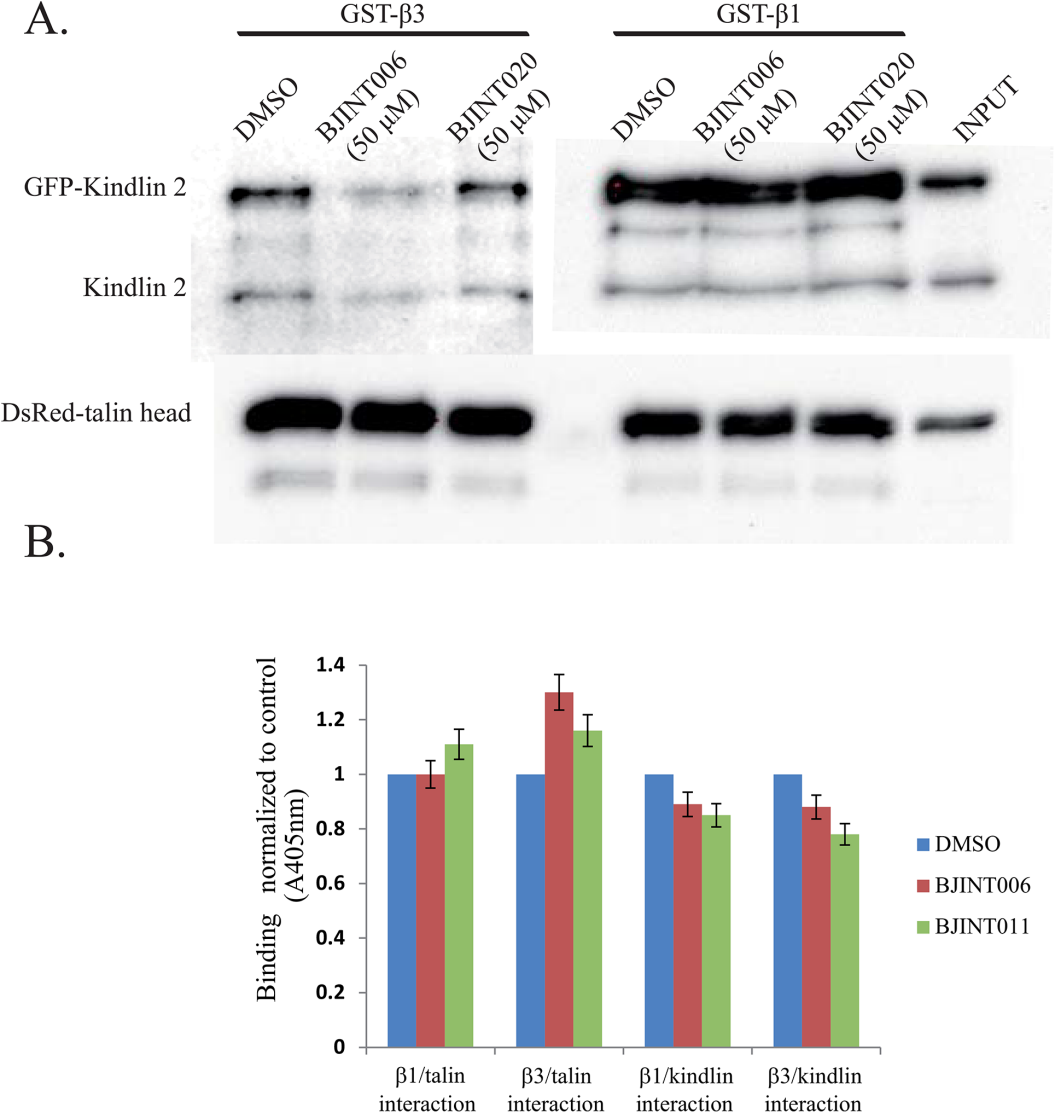
BJINT derivatives interfere with the binding of talin and kindlin to integrin cytoplasmic tails. (A) Pull-down assays with GST-β_3_ and GST-β_1_ tails bound to glutathione beads incubated for 4 h with cells lysates. Associated talin head and kindlin was revealed Western blotting using anti kindlin and anti talin head antibodies. Chemiluminescence was monitored by Biorad imager. (B) Summary of 6 solid phase binding assays of biotinylated integrin tails to talin (F2/F3 domain) and kindlin-2 (FERM domain) under non saturable conditions and in the presence of 50 μM BJINT derivatives.

Talin and kindlin recruitment onto the β cytoplasmic tail is regarded as the end point of integrin activation [34]. Therefore we studied the possible competition between the small molecules and these proteins for the interactions with the integrin intracellular domain. Pull-down assays were carried out to investigate the interaction between overexpressed DsRed-talin head domain or endogenous kindlin-2, overexpressed GFP-kindlin-2, and GST-β_3_ or GST-β_1_ cytoplasmic domains in presence or absence of BJINT006 or 020. Under our experimental conditions, no differences could be observed in talin head/β_3_ or β_1_ integrin interactions whatever the drug used, while kindlin-2 interaction with the β_3_ cytoplasmic domain but not β_1_ was reduced up to 70% by BJINT 006, but not BJINT020, consistent with *ex vivo* results (Fig 5A). However, in pull down assays, molar ratios between integrins and cytoplasmic partners are not controlled and we could not exclude that under our experimental conditions, drug inhibition on β_1_ interactions with its partners might have been blunted by an excess of ligand. In addition, since the drugs were added into the cytosol, one cannot exclude an additional effect of these drugs onto an upstream or alternative regulatory mechanisms of talin and kindlin recruitment. Therefore, we designed a solid phase binding assay of purified biotinylated integrin tails fused to GST onto immobilized purified GST tagged kindlin-2 FERM domain or talin F2/F3 domain. Unspecific binding was estimated using plain GST. This assay allowed the measurement of typical saturation curves (S4 Fig) and to determine the integrin tail concentrations under which the interaction with the partner should be sensitive to a competitive inhibitors. Under these experimental conditions, the overall drug inhibition of the binding of kindlin-2 FERM domain or of talin F2/F3 domain on β_1_ or β_3_ tails were either absent or quite small even at 50 μM, (Fig 5B). NMR studies to detect a direct interaction of BJINT 006 on the β_3_ cytoplasmic domain exhibited very small shifts that were identical for all amino acids, suggesting a non-specific interaction (S6 Fig). On the other hand, ITC experiments did not reveal any interaction (not shown). Altogether, these data suggested that BJINT compounds may not specifically interact with integrin tails. Therefore one could conclude that BJINT molecules interfere with integrin activation events upstream or alternative to talin and kindlin recruitment.

### BJINT derivatives inhibit outside-in integrin signaling

**Fig 6:**
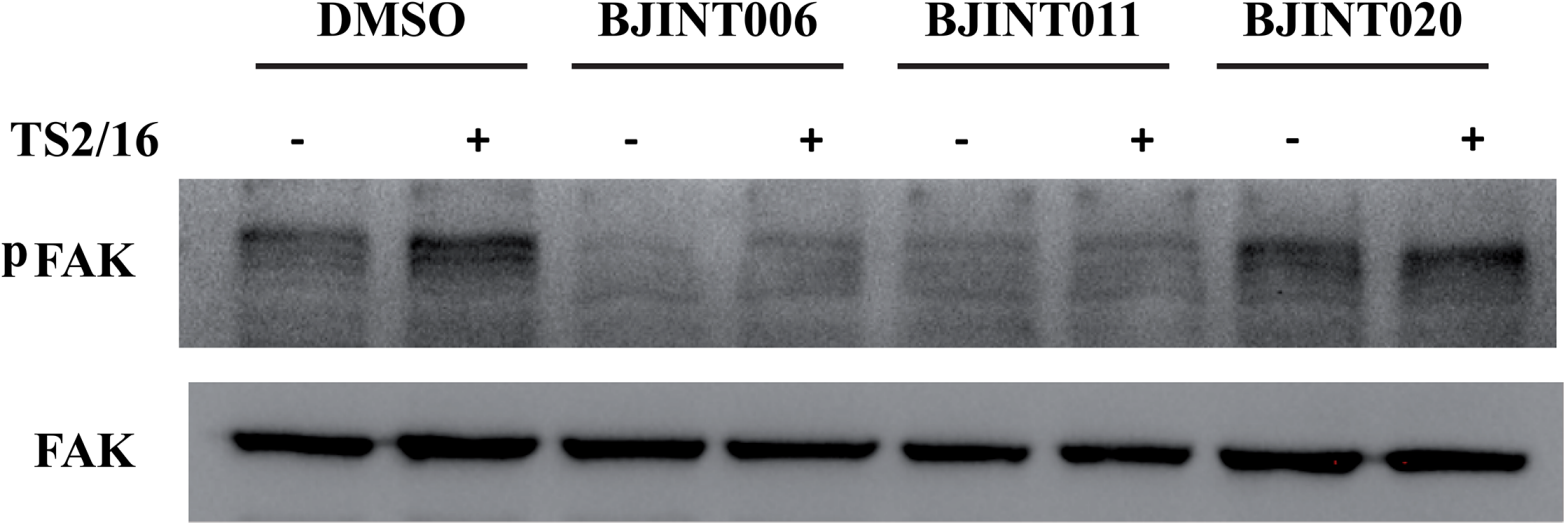
BJINT derivatives blunt integrin outside-in signaling. Auto-phosphorylation of FAK, one of the earliest integrin dependent signaling events was used as readout of integrin signaling activity. 10^7^ HeLa cells were harvested and incubated for 1 h at 37°C in D-MEM, then suspended at the concentration of 10^6^ cells/mL as described in [35] with TS2/16 monoclonal antibody and BJINT molecules or DMSO. Cells were centrifuged and lysed in Laemmli's sample buffer and analyzed by Western blotting.

Many biases with currently available integrin antagonists originate from their ability to trigger outside-in signaling while they efficiently inhibit inside-out signaling and subsequent cell-matrix or cell-cell interactions. Since BJINT derivatives target integrin tails, we wondered whether they were able to hamper integrin outside-in signaling. As read-out we looked at the auto-phosphorylation of FAK, one of the earliest events of integrin signaling using the established procedure described in [35]. Briefly, HeLa cells were re-suspended in the medium to switch off integrin signaling, then specific integrin signaling was switched on again by adding the activating β_1_ integrin monoclonal antibody TS2/16 in presence or absence of the drug. In that way, the action of BJINT molecules could not be attributed to an indirect effect due to cell detachment. After one hour in suspension, phosphorylation of tyrosine 397 still could be detected in cell lysates, although this level was slightly increased upon addition of the β_1_ activating monoclonal antibody TS2/16. BJINT006 and 011, but not 020, completely abolished FAK auto-phosphorylation and likely all the downstream stages of integrin signaling (Fig 6).

## Discussion

The data presented indicate that the previously described inhibition of cell migration by 3-arylquinoline and 3-aryl-2-quinolone derivatives was likely due to the ability of these compounds to alter the integrity of structures relying on integrins, as visualized by GFP-kindlin-2 delocalization. Conversely to Kindlin-1 and -3, kindlin-2 is universally expressed and constitutes a choice marker of focal adhesions whatever the cell line used. Since integrin activation was largely described to be dependent on the recruitment of kindlin-2 [36, 37], delocalization of GFP-kindlin-2 appeared as a pertinent read-out. Kindlin-3 is preferentially expressed in blood cell lineage. A decrease in its expression in humans causes type III leukocyte adhesion deficiency (LAD-III), which is associated with an inability to activate integrins on platelets and leukocytes and manifests as susceptibility to bleeding and infections. However, kindlin-2 was shown to be able to activate β_3_ integrins at least ex vivo [38], indicating that both proteins have a similar function regarding β_3_ integrin activation. Conversely, kindlin-3 was shown to be unable to compensate kindlin-2 loss for α_5_β_1_ activation in fibroblasts [39].

Our experiments ruled out the possibility that BJINT inhibition of cell adhesion was directly linked to cell contractility, FAK, and Src signaling. In addition, the antagonistic action of Mn^2+^ (a specific activator of integrin receptors) together with the drug insensibility of cells attached on Poly-lysine point to the targeting of integrin activation by BJINT through an indirect but common process on inside-out mechanism resulting in the lack of specificity toward extracellular matrix components. In turn, the switch to the integrin low affinity conformation would result in the disruption of the adhesive structures.

This indirect effect is suggested by the lack of any detected interaction of BJINT molecules with integrin tails and the absence of an inhibitory effect of the drugs on the binding of the purified tails to purified integrin partners such as kindlins and talin, although we cannot rule out the possibility that full length talin or kindlin-2 may behave differently than the F2/F3 talin domain and kindlin-2 FERM domain, respectively. In addition, since both pull downs and solid phase assays were carried out in detergent to minimize non-specific binding, the hydrophobic nature of these small molecules may account for their trapping into micelles, thus resulting in a strong decrease in their actual efficient concentration. In vivo, membranes play a major role in integrin activation and their interaction with cytoplasmic partners, and may also strongly modulate BJINT inhibitory activity. For instance, under physiological conditions, not only talin head domain, but also talin tail may participate to the integrin activation process, which also requires the disruption of the saline bridge and separation of α and β cytoplasmic domains. In addition, it was recently reported that talin interaction with β_3_ tail occurs in two waves, one which triggers external ligand binding but is not involved in outside-in signaling the other one that is dependent on Gα13 which selectively mediates outside–in signaling [40]. Therefore it is conceivable that BJINT molecules only impair this second interacting process while pull down assays only monitor the first one. Phosphoinositide phosphate have also been reported to play a role in kindlin-2 activity [41]. Finally, while evidence has recently been provided that talin head and kindlin can interact at the same time on integrin tail [42], the spatiotemporal roles of these two major players are yet to be unraveled.

Upstream or alternative direct targets of BJINT derivatives can be envisioned. For instance, kindlin-2 biding to Integrin linked kinase (ILK) pseudokinase complex was reported to play a major role in focal adhesion localization of the protein [43]. Other possible candidates are adaptors proteins allowing the membrane recruitment of the Rap1-interacting adapter molecule (RIAM) as already demonstrated for α_IIb_β_3_ integrins [44]. Finally, the drug may strengthen the interaction with endogenous inhibitors in the α cytoplasmic tail such as sharpin [45].

Whatever the mechanism, it is clear that BJINT molecules blunt both inside-out and outside-in integrin signaling with a broad receptor spectra. As expected for such a mechanism, BJINT derivatives block the integrin dependent processes in platelets, and are therefore potential anti-thrombotic agents. For ADP triggered aggregation however, a limited initial phase of aggregation was still present. Recently it has been proposed that α_IIb_β_3_ activation is triggered by talin while kindlin favors clustering [21]. According to this mechanism, and since BJINT derivatives seem to favor rather than inhibit talin/β_3_ and blocks kindling β_3_ tail interaction, it is conceivable that they only block the integrin clustering required for sustaining platelet aggregation over time.

SAR studies with twenty members of the 3-arylquinoline and 3-aryl-2-quinolone series shed some light on important structural features required for the improvement of the molecule efficiency. Higher affinity compounds will allow structural studies that should help optimizing the BJINT family members to reach the submicromolar affinity, thus opening the doors to therapeutic uses. By blocking the integrin cytoplasmic face, this new class of molecules is expected to minimize integrin outside-in signaling that is a major drawback of all RGD-mimetic agents presently used in therapy. Inhibiting integrin signaling has long been thought to be a potent way to suppress unwanted downstream signaling pathways [3]. The proof of concept that such a strategy can be achieved has been provided a couple of years ago when α_4_-integrin/paxillin interaction has been inhibited by a small molecule (Kummer et al., 2010). Herein, we showed using FAK auto-phosphorylation as readout that bioactive BJINT derivatives efficiently blunt integrin outside-in signaling.

Our work provides the first example of small molecules able to cross the plasma membrane and impair integrin both inside-out and outside-in signaling. It demonstrates that 3-arylquinoline and 3-aryl-2-quinolone derivatives can be efficiently used to block a physiological process, i.e. platelet activation.

## Supporting Information

S1 Fig Colocalization of GFP-kindlin-2 and kindlin-2. (EPS)

S2 Fig Toxicity of BJINT derivatives: Analyses of 3 representative molecules. (EPS)

S3 Fig BJINT006 cell adhesion inhibition is not specific to an ECM substrate. (EPS)

S4 Fig Synthesis strategy for BJINT010-BJINT019. (EPS)

S5 Fig Binding of β_1_ and β_3_ integrin tails onto talin F2/F3 domain and kindlin-2 FERM domain. (EPS)

S6 Fig RMN signal shifts with β3 cytoplasmic tail in the presence or absence of BJINT 06 (EPS)

S1 Methods Chemical general procedure, synthesis and characterizations. (DOCX)
