# Supplementary material for: Targeting Integrin-Dependent Adhesion and Signaling with 3-Arylquinoline and 3-Aryl-2-Quinolone Derivatives: A new Class of Integrin Antagonists
Source: PLoS One. 2015 Oct 28;10(10):e0141205. doi: 10.1371/journal.pone.0141205 (PMC4624933; doi:10.1371/journal.pone.0141205)
Supplement: S1 Methods — (DOCX) [file pone.0141205.s007.docx]

# **Chemistry General Procedures**

Commercial reagents (Fluka, Aldrich) were used without purification. Solvents were distillated prior to use. All reactions requiring anhydrous conditions were conducted in oven-dried apparatus. Melting points were determined using a Büchi capillary instrument and are uncorrected. IR spectra were recorded on a Perkin-Elmer 681 infrared spectrophotometer. ^1^H NMR spectra were recorded on a Bruker Avance 300 MHz spectrometer. Chemical shifts are reported in ppm (δ) and were referenced to DMSO-*d_6_* (2.50 ppm) or CDCl3 (7.26 ppm). Mass spectra were recorded with a Perkin-Elmer SCIEX API spectrometer. Elemental analyses were performed on a Thermoquest Flash 1112 series EA analyzer. Thin Layer Chromatography (TLC) analyses were conducted on aluminium sheets silica gel Merck 60F_254_. The spots were visualized using an ultraviolet light. Flash chromatography was carried out on silica gel 60 (40-63 µm, Merck) using the indicated solvents. The light petroleum ether refers to the fraction boiling at 40-60°C. Compound 1 (BJINT020), BJINT001, BJINT002, BJINT003, BJINT004, BJINT005, BJINT006, BJINT007, BJINT008 and BJINT009 were synthesized as described previously (Joseph, et al., 2002) and provided by Benoît Joseph’s research group. Other BJINT compounds were synthetized as described on Figure S6.

**2-{4-[5,7-Dimethoxy-3-(4-methoxyphenyl)-2-oxo-2*H*-quinolin-1-yl]butyl}isoindole-1,3-dione (2)** - Under argon atmosphere, sodium hydride (300 mg, 12.5 mmol, 60% oil dispersion) was added portionwise to a solution of 5,7-dimethoxy-3-(4-methoxyphenyl-1,2-dihydro-2-quinolone **1** (1.5 g, 4.8 mmol) in dry DMF (30 mL) at 0°C. The mixture was stirred for 15 min at 0°C, and then *N*-(4-bromobutyl)phthalimide (3.12 g, 11.0 mmol) in dry THF/DMF (3:1, 40 mL) was added. The final mixture was stirred at 90°C for 18 h. The solvent was evaporated and the crude residue was partitioned between H_2_O (20 mL) and CH_2_Cl_2_ (20 mL). After extraction, the organic layers were dried over MgSO_4_ and concentrated in vacuo. The residue was purified by column chromatography (eluent petroleum ether/EtOAc 2:1 then 1:1) to give **2** (758 mg, 38%). Mp = 171-172 °C. ^1^H NMR (300 MHz, DMSO-*d*_6_): δ 1.70 (broad s, 4H, CH_2_), 3.63 (t, 2H, *J* = 6.0 Hz, CH_2_), 3.78 (s, 3H, OCH_3_), 3.87 (s, 3H, OCH_3_), 3.91 (s, 3H, OCH_3_), 4.40 (t, 2H, *J* = 6.0 Hz, CH_2_), 6.44 (d, 1H, *J* = 1.5 Hz, H_Ar_), 6.54 (broad s, 1H, H_Ar_), 6.95 (d, 2H, *J* = 8.8 Hz, H_Ar_), 7.61 (d, 2H, *J* = 8.8 Hz, H_Ar_), 7.83 (broad s, 4H, H_Ar_), 7.97 (s, 1H, H_Ar_). MS (IS): *m/z* 513 (M + H^+^). Anal. calcd for C_30_H_28_N_2_O_6_: C, 70.30; H, 5.51; N, 5.47. Found: C, 70.05; H, 5.45; N, 5.56.

**1-(4-Aminobutyl)-5,7-dimethoxy-3-(4-methoxyphenyl)-1*H*-quinolin-2-one (3)** - A solution of **2** (348 mg, 0.68 mM) and hydrazine hydrate (43.0 µL, 0.83 mmol) in EtOH (7 mL) was stirred at reflux overnight. The solvent was evaporated and the crude residue was taken up in 1N HCl (20 mL) and washed with EtOAc (2 x 20 mL). The aqueous layer was basified with 1N NaOH (pH=10) and extracted with EtOAc (4 x 20 mL). The organic layers were dried over MgSO_4_ and concentrated in vacuo to give **3** (238 mg, 92%). The compound was used without further purification. Mp = 190-192°C (EtOAc). ^1^H NMR (300 MHz, DMSO-*d*_6_): δ 1.43-1.52 (m, 2H, CH_2_), 1.64-1.74 (m, 2H, CH_2_), 2.62 (t, 2H, *J* = 6.8 Hz, CH_2_), 3.79 (s, 3H, OCH_3_), 3.92 (s, 3H, OCH_3_), 3.93 (s, 3H, OCH_3_), 4.28 (t, 2H, *J* = 7.5 Hz, CH_2_), 4.35 (broad s, 2H, NH_2_), 6.49 (d, 1H, *J* = 1.7 Hz, H_Ar_), 6.61 (broad s, 1H, H_Ar_), 6.96 (d, 2H, *J* = 8.8 Hz, H_Ar_), 7.63 (d, 2H, *J* = 8.8 Hz, H_Ar_), 8.00 (s, 1H, H_Ar_). MS (IS): *m/z* 383 (M + H^+^). Anal. Calcd for C_22_H_26_N_2_O_4_: C, 69.09; H, 6.85; N, 7.32. Found: C, 68.91; H, 6.86; N, 7.36.

**1-(4-Dimethylaminobutyl)-5,7-dimethoxy-3-(4-methoxyphenyl)-1*H*-quinolin-2-one (BJINT010)** - A solution of **3** (225 mg, 0.59 mM), 37% HCHO in H_2_O (0.18 mL, 2.36 mmol) and AcOH (0.18 mL, 4.72 mmol) in MeOH (3 mL) was stirred at reflux overnight. After cooling, the medium was acidified until pH=1 with 10N HCl and washed with EtOAc. The aqueous layer was basified with 5N NaOH (pH=10) and extracted with CH_2_Cl_2_ (3 x 20 mL). The organic layers were dried over MgSO_4_ and concentrated in vacuo to give **BJINT010** (194 mg, 80%). Oil. ^1^H NMR (300 MHz, CDCl_3_): δ 1.65-1.73 (m, 2H, CH_2_), 1.78-1.90 (m, 2H, CH_2_), 2.30 (s, 6H, NCH_3_), 2.42 (t, 2H, *J* = 7.2 Hz, CH_2_), 3.83 (s, 3H, OCH_3_), 3.91 (s, 3H, OCH_3_), 3.92 (s, 3H, OCH_3_), 4.32 (t, 2H, *J* = 7.6 Hz, CH_2_), 6.29 (d, 1H, *J* = 1.9 Hz, H_Ar_), 6.45 (d, 1H, *J* = 1.9 Hz, H_Ar_), 6.94 (d, 2H, *J* = 8.8 Hz, H_Ar_), 7.68 (d, 2H, *J* = 8.8 Hz, H_Ar_), 8.13 (s, 1H, H_Ar_). MS (IS): *m/z* 411 (M + H^+^). Anal. Calcd for C_24_H_30_N_2_O_4_: C, 70.22; H, 7.37; N, 6.82. Found: C, 70.33; H, 7.40; N, 6.77.

**1-(4-Diethylaminopropyl)-5,7-dimethoxy-3-(4-methoxyphenyl)-1*H*-quinolin-2-one (BJINT011)** - According to the procedure described for the synthesis of **2**, N-alkyl and O-alkyl compounds **BJINT011** and **BJINT012** were prepared from **1** (1 equivalent) and 3-(diethylamino)propyl chloride (2 equivalents). Reaction time: 3 h. Eluent chromatography: Et_2_O/MeOH 8:2 and then CH_2_Cl_2_/MeOH 9:1. Yield: 44%. Oil. ^1^H NMR (300 MHz, CDCl_3_): δ 1.35 (t, 6H, *J* = 7.3 Hz, CH_3_), 2.40-2.50 (m, 2H, CH_2_), 3.06-3.20 (m, 6H, CH_2_), 3.83 (s, 3H, OCH_3_), 3.92 (s, 3H, OCH_3_), 4.00 (s, 3H, OCH_3_), 4.42 (t, 2H, *J* = 7.0 Hz, CH_2_), 6.31 (d, 1H, *J* = 1.9 Hz, H_Ar_), 6.46 (d, 1H, *J* = 1.9 Hz, H_Ar_), 6.95 (d, 2H, *J* = 8.8 Hz, H_Ar_), 7.63 (d, 2H, *J* = 8.8 Hz, H_Ar_), 8.16 (s, 1H, H_Ar_). MS (IS): *m/z* 425 (M + H^+^). Anal. Calcd for C_25_H_32_N_2_O_4_: C, 70.73; H, 7.60; N, 6.60. Found: C, 70.62; H, 7.55; N, 6.71.

**{3-[5,7-Dimethoxy-3-(4-methoxyphenyl)quinolin-2-yloxy]propyl}diethylamine (BJINT012) -** Yield: 7%. Mp = 52-54°C. ^1^H NMR (300 MHz, CDCl_3_): δ 1.04 (t, 6H, *J* = 7.3 Hz, CH_3_), 1.95-2.05 (m, 2H, CH_2_), 2.50-2.75 (m, 6H, CH_2_), 3.85 (s, 3H, OCH_3_), 3.93 (s, 3H, OCH_3_), 3.94 (s, 3H, OCH_3_), 4.51 (t, 2H, *J* = 6.2 Hz, CH_2_), 6.39 (d, 1H, *J* = 2.1 Hz, H_Ar_), 6.81 (d, 1H, *J* = 2.1 Hz, H_Ar_), 6.95 (d, 2H, *J* = 8.8 Hz, H_Ar_), 7.57 (d, 2H, *J* = 8.8 Hz, H_Ar_), 8.25 (s, 1H, H_Ar_). MS (IS): *m/z* 425 (M + H^+^). Anal. Calcd for C_25_H_32_N_2_O_4_: C, 70.73; H, 7.60; N, 6.60. Found: C, 70.67; H, 7.62; N, 6.67.

**1-(4-Dimethylaminopropyl)-5,7-dimethoxy-3-phenyl-1*H*-quinolin-2-one (BJINT013) -** According to the procedure described for **2**, N-alkyl and O-alkyl compounds **BJINT013** and **BJINT014** were prepared from 5,7-dimethoxy-3-phenyl-1,2-dihydro-2-quinolone **4** (Joseph et al., 2002) (1 equivalent) and 3-(dimethylamino)propyl chloride (2 equivalents). Reaction time: 3 h. Eluent chromatography: Et_2_O/MeOH 8:2 and then CH_2_Cl_2_/MeOH 9:1. Yield: 51%. Mp = 90-92°C (Et_2_O washing). ^1^H NMR (300 MHz, CDCl_3_): δ 1.95-2.10 (m, 2H, CH_2_), 2.34 (s, 6H, NCH_3_), 2.54 (t, 2H, *J* = 7.3 Hz, CH_2_), 3.93 (s, 6H, OCH_3_), 4.38 (t, 2H, *J* = 7.3 Hz, CH_2_), 6.30 (d, 1H, *J* = 1.9 Hz, H_Ar_), 6.54 (s, 1H, H_Ar_), 7.29-7.43 (m, 3H, H_Ar_), 7.71-7.74 (m, 2H, H_Ar_), 8.19 (s, 1H, H_Ar_). MS (IS): *m/z* 367 (M + H^+^). Anal. Calcd for C_22_H_26_N_2_O_3_: C, 72.11; H, 7.15; N, 7.64. Found: C, 71.88; H, 7.02; N, 7.50.

**{3-[5,7-Dimethoxy-3-phenylquinolin-2-yloxy]propyl}dimethylamine (BJINT014) -** Yield: 9%. Mp = 102-104°C (Et_2_O washing). ^1^H NMR (300 MHz, CDCl_3_): δ 1.95-2.05 (m, 2H, CH_2_), 2.27 (s, 6H, NCH_3_), 2.48 (t, 2H, *J* = 7.1 Hz, CH_2_), 3.93 (s, 6H, OCH_3_), 4.53 (t, 2H, *J* = 6.4 Hz, CH_2_), 6.40 (d, 1H, *J* = 2.1 Hz, H_Ar_), 6.82 (d, 1H, *J* = 2.1 Hz, H_Ar_), 7.31-7.45 (m, 3H, H_Ar_), 7.60-7.65 (m, 2H, H_Ar_), 8.30 (s, 1H, H_Ar_). MS (IS): *m/z* 367 (M + H^+^). Anal. Calcd for C_22_H_26_N_2_O_3_: C, 72.11; H, 7.15; N, 7.64. Found: C, 72.33; H, 7.17; N, 7.52.

**1-(4-Dimethylaminopropyl)-7-methoxy-3-(4-methoxyphenyl)-1*H*-quinolin-2-one (BJINT015) -** According to the procedure described for **2**, N-alkyl and O-alkyl compounds **BJINT015** and **BJINT016** were prepared from 7-methoxy-3-(4-methoxyphenyl)-1,2-dihydro-2-quinolone **5** (Mehta et al., 2010) (1 equivalent) and 3-(dimethylamino)propyl chloride (2 equivalents). Reaction time: 30 h. Eluent chromatography: Et_2_O/MeOH 8:2 and then CH_2_Cl_2_/MeOH 9:1. Yield : 45%. Mp = 45-47°C. ^1^H NMR (300 MHz, CDCl_3_): δ 1.95-2.05 (m, 2H, CH_2_), 2.32 (s, 6H, NCH_3_), 2.52 (t, 2H, *J* = 7.1 Hz, CH_2_), 3.84 (s, 3H, OCH_3_), 3.93 (s, 3H, OCH_3_), 4.53 (t, 2H, *J* = 7.3 Hz, CH_2_), 6.83 (dd, 1H, *J* = 2.1, 8.5 Hz, H_Ar_), 6.94 (broad s, 1H, H_Ar_), 6.96 (d, 2H, *J* = 8.8 Hz, H_Ar_), 7.51 (d, 1H, *J* = 8.5 Hz, H_Ar_), 7.66 (d, 2H, *J* = 8.8 Hz, H_Ar_), 7.68 (s, 1H, H_Ar_). MS (IS): *m/z* 367 (M + H^+^). Anal. Calcd for C_22_H_26_N_2_O_3_: C, 72.11; H, 7.15; N, 7.64. Found: C, 72.10; H, 7.10; N, 7.67.

**1-(4-Dimethylaminobutyl)-5,7-dimethoxy-3-phenyl-1*H*-quinolin-2-one (BJINT016) -** Yield: 8%. Oil. ^1^H NMR (300 MHz, CDCl_3_): δ 1.98-2.07 (m, 2H, CH_2_), 2.29 (s, 6H, NCH_3_), 2.51 (t, 2H, *J* = 7.0 Hz, CH_2_), 3.87 (s, 3H, OCH_3_), 3.94 (s, 3H, OCH_3_), 4.55 (t, 2H, *J* = 6.4 Hz, CH_2_), 6.97 (d, 2H, *J* = 8.8 Hz, H_Ar_), 7.03 (dd, 1H, *J* = 2.3, 8.8 Hz, H_Ar_), 7.21 (d, 1H, *J* = 2.3 Hz, H_Ar_), 7.56 (d, 2H, *J* = 8.8 Hz, H_Ar_), 7.61 (d, 1H, *J* = 8.8 Hz, H_Ar_), 7.88 (s, 1H, H_Ar_). MS (IS): *m/z* 367 (M + H^+^). Anal. Calcd for C_22_H_26_N_2_O_3_: C, 72.11; H, 7.15; N, 7.64. Found: C, 72.37; H, 7.28; N, 7.51.

**{4-[5,7-Dimethoxy-3-(4-methoxyphenyl)quinolin-2-yloxy]butyl}dimethylamine (BJINT017)** - To a solution of 4-dimethylaminobutanol (0.15 mL, 1.13 mmol) in DMF (7.5 mL) was added sodium (21 mg, 0.91 mmol) at 0°C. The mixture was warmed to room temperature and after complete consumption of sodium, copper (24 mg, 0.91 mmol) and 2-chloro-5,7-dimethoxy-(4-methoxyphenyl)quinoline **6** ([Croisy, Huel, & Bisagni, 1997](file:///C:\Users\Benoit\Documents\BJ%20SAUV%2010-13\BJ%20bureau%2010-13\ACSChemBiol2013\PNAS\article-text_OVBJ.docx#_ENREF_7)) (123 mg, 0.37 mmol) were added. The final mixture stirred at 110°C for 40 h. After evaporation of the solvent, the crude residue was purified by column chromatography (eluent CH_2_Cl_2_/MeOH 97:3 and then 9:1) to give **BJINT017** (15 mg, 10%). Oil. ^1^H NMR (300 MHz, CDCl_3_): δ 1.55-1.90 (m, 4H, CH_2_), 2.42 (s, 6H, NCH_3_), 2.64 (, 2H, CH_2_), 3.85 (s, 3H, OCH_3_), 3.94 (s, 6H, OCH_3_), 4.51 (t, 2H, *J* = 4.7 Hz, CH_2_), 6.40 (d, 1H, *J* = 2.1 Hz, H_Ar_), 6.81 (d, 1H, *J* = 2.1 Hz, H_Ar_), 6.96 (d, 2H, *J* = 8.8 Hz, H_Ar_), 7.54 (d, 2H, *J* = 8.8 Hz, H_Ar_), 8.25 (s, 1H, H_Ar_). MS (IS): *m/z* 411 (M + H^+^). Anal. Calcd for C_24_H_30_N_2_O_4_: C, 70.22; H, 7.37; N, 6.82. Found: C, 70.04; H, 7.55; N, 6.88.

{**3-[5,7-Dimethoxy-(4-methoxyphenyl)quinolin-2-yl]prop-2-ynyl}dimethylamine (BJINT018)** - A solution of **6** (85 mg, 0.26 mM), N,N-dimethylprop-2-ynylamine (139 µL, 1.30 mmol), PdCl_2_(PPh_3_)_2_ (18 mg, 0.03 mmol), CuI (5 mg, 0.03 mmol) and Et_3_N (144 µL, 1.04 mmol) in DMF (2 mL) was stirred at 60°C for 5 h. The solution was diluted by addition of CH_2_Cl_2_ (20 mL), washed with 2% NH_4_OH (20 mL) and brine. The organic layer was dried over Na_2_SO_4_ and concentrated in vacuo. The residue was purified by column chromatography (eluent Et_2_O/MeOH 8:2) to give **BJINT018** (91 mg, 94%). Mp = 70-72°C (EtOAc/PE). ^1^H NMR (300 MHz, CDCl_3_): δ 2.20 (s, 6H, NCH_3_), 3.48 (s, 2H, CH_2_), 3.85 (s, 3H, OCH_3_), 3.92 (s, 3H, OCH_3_), 3.95 (s, 3H, OCH_3_), 6.50 (d, 1H, *J* = 2.1 Hz, H_Ar_), 6.96 (d, 2H, *J* = 8.8 Hz, H_Ar_), 7.03 (d, 1H, *J* = 2.1 Hz, H_Ar_), 7.56 (d, 2H, *J* = 8.8 Hz, H_Ar_), 8.35 (s, 1H, H_Ar_). MS (IS): *m/z* 377 (M + H^+^). Anal. Calcd for C_23_H_24_N_2_O_3_: C, 73.38; H, 6.43; N, 7.44. Found: C, 73.57; H, 6.27; N, 7.51C, H, N.

**{3-[5,7-Dimethoxy-3-(4-methoxyphenyl)quinolin-2-yl]propyl}dimethylamine (BJINT019)** - To a solution of **BJINT018** (80 mg, 0.21 mmol) in EtOH (3 mL) was added 10% Pd/C (20 mg). The final mixture was stirred overnight under 5 bars of hydrogen at room temperature. The catalyst was removed by filtration over Celite (MeOH elution). After evaporation of the solvent, the residue was purified by column chromatography (CH_2_Cl_2_/MeOH 95:5 to 9:1) to give **BJINT019** (66 mg, 82%). Mp = 82-84°C. ^1^H NMR (300 MHz, CDCl_3_): δ 2.00-2.11 (m, 2H, CH_2_), 2.45 (s, 6H, NCH_3_), 2.64 (t, 2H, *J* = 7.7 Hz, CH_2_), 2.95 (t, 2H, *J* = 7.5 Hz, CH_2_), 3.87 (s, 3H, OCH_3_), 3.94 (s, 3H, OCH_3_), 3.95 (s, 3H, OCH_3_), 6.49 (d, 1H, *J* = 2.1 Hz, H_Ar_), 6.97 (broad s, 1H, H_Ar_), 6.98 (d, 2H, *J* = 8.8 Hz, H_Ar_), 7.28 (d, 2H, *J* = 8.8 Hz, H_Ar_), 8.21 (s, 1H, H_Ar_). MS (IS): *m/z* 381 (M + H^+^). Anal. Calcd for C_23_H_28_N_2_O_3_: C, 72.61; H, 7.42; N, 7.36. Found: C, 72.69; H, 7.45; N, 7.40.
